# Ablative Therapies for Colorectal Polyps and Malignancy

**DOI:** 10.1155/2014/986352

**Published:** 2014-06-26

**Authors:** Jacqueline Oxenberg, Steven N. Hochwald, Steven Nurkin

**Affiliations:** Roswell Park Cancer Institute, Elm & Carlton Streets, Buffalo, NY 14263, USA

## Abstract

Endoscopic techniques are gaining popularity in the management of colorectal polyps and occasionally superficial cancers. While their use is in many times palliative, they have proven to be curative in carefully selected patients with polyps or malignancies, with less morbidity than radical resection. However, one should note that data supporting local and ablative therapies for colorectal cancer is scarce and may be subject to publication bias. Therefore, for curative intent, these techniques should only be considered in highly select cases as higher rates of local recurrences have also been reported. The aim of this review is to explain the different modalities of local and ablative therapies specific to colorectal neoplasia and explain the indications and circumstances where they have been most successful.

## 1. Introduction

Ablative therapies for polyps and malignancies of the colon and rectum were first described decades ago but modalities have since evolved as new technologies emerge. Choosing ablation over resection for therapeutic benefit should be highly selective, especially for high risk polyps and early malignancies. While these techniques may be less invasive and possibly less morbid than surgical resections, the tradeoff is lack of pathologic assessment and risk of residual or recurrent disease. One should remember that cellular architecture of tissue is assessed only by biopsy or excision and not by tumor destruction, which limits the ability to assess aggressive cellular features, margin status, and stage. Therefore, ablative therapies for cure of malignancies should rarely be used.

Techniques such as endoscopic mucosal resections (EMR) and endoscopic submucosal dissections (ESD) are becoming more common for resection of higher risk polyps or superficial cancers. With the use of endoscopic resection, the clinicopathologic features of malignant polyps, including depth of tumor invasion, angiolymphatic invasion, degree of differentiation, and margins of resection, can help identify those patients at risk for harboring nodal metastases [1–5]. Using these features, further surgical recommendations are guided by the risk of both locoregional and distant recurrences [6, 7]. In low risk polyps, endoscopic resection can be adequate and frequently curative [8–10]. Indications for endoscopic resections of colorectal cancers for cure are highly selective and according to Japanese Consensus Guidelines should only be performed for lesions with the following characteristics: low risk for lymph node metastases, intramucosal or have slight submucosal invasion, diameter less than 2 cm, and macroscopic features [11]. The National Comprehensive Cancer Network similarly supports endoscopically removed or transanally excised malignant polyps as a sole treatment in patients only with favorable histopathologic features such as <3 cm, T1, grade 1 or grade 2, no lymphatic or venous invasion, and negative margins, although sessile polyp configuration is controversial [8, 9, 12, 13].

Because treatments for rectal cancer are typically aggressive and include morbid surgical resections, patient goals and tumor characteristics should help guide treatment recommendations. For these reasons, interest in less invasive treatments, such as transanal excision, transanal endoscopic microsurgery (TEM), or similarly transanal minimally invasive surgery (TAMIS), especially for early staged rectal cancers, is increasing. Transanal excision and TEM involve a full-thickness excision performed through the bowel wall and into the perirectal fat. For mucosal lesions, a resection within the submucosal plane may also be performed. Advantages include minimal morbidity and mortality and rapid postoperative recovery [14]. Patients inclined to receive transanal resections over radical resections include those unfit for major surgery, those who refuse major surgery, or those who have a small primary tumor with low risk of nodal or distant metastases as stated above [15]. Tumor characteristics amenable to local therapies typically include mobile (confined to the bowel wall), ≤3 cm in diameter, and located below the peritoneal reflection [15].

Ablative therapies may have a role for patients with benign as well as malignant colorectal diseases. Ablation may be used for margin attenuation after polypectomy. In addition, palliation using ablation for symptoms should be entertained in patients that are considered noncurative. Ablative therapies for symptoms of obstruction, bleeding, tenesmus, pain, diarrhea, and others are reported. More aggressive surgical procedures such as a diverting stoma, segmental colon resection, or stent placement can also be performed with reasonable outcomes and improvement in quality of life.

## 2. Ablative Techniques

### 2.1. Electrocoagulation or Fulguration

Electrocoagulation or fulguration was first described by Byrne in 1889 for the treatment of gynecologic malignancies but was popularized for rectal tumors by Strauss et al. in 1935 [16]. It involves transanal exposure of the tumor, usually with a proctoscope, applying needle-tip monopolar or bipolar cautery into and through the tumor as well as a 1 cm halo circumferentially with charred tumor removed and repeated until no further tumor is seen [15]. It can be performed in the lithotomy position under light general anesthesia with a local anesthetic as well as 1 : 200,000 epinephrine. Posteriorly, electrocoagulation can be performed aggressively to the presacral fascia but coagulation of anterior lesions should be performed more cautiously due to a higher perforation risk [17]. Questionable areas should be rebiopsied and electrocoagulation can be repeated, but radical resection should be performed if multiple procedures are necessary or hard satellite nodes are found [17].

Electrocautery/fulguration of colorectal malignancies for curative intent has been reported in a number of studies. The largest reported series from Madden et al. included 204 patients with tumors up to 8 cm and few involved the full circumference of the rectal wall. No local or distant disease was found in 62%; overall 5-year survival was 72% and morbidity was 23.5% with bleeding most common [18]. In a series of 81 patients treated for curative intent, overall complication rate was 21% and included bleeding (7%), stricture (6%), urinary retention (2.6%), electrical burns (2.6%), perianal abscess (0.9%), and perforation (0.9%). Unfortunately, 38% eventually required abdominoperineal resection and overall 5-year survival was 58% in patients treated solely with fulguration [19]. In another series, of 39 patients treated for curative intent, 5 (13%) required APR for disease control. All but 2 patients had moderate to well-differentiated tumors and, of the 27 patients without evidence of tumor at end of followup, only 9 required a single treatment. Success of treatment was significantly improved for polypoid/exophytic (92%) tumors compared to ulcerative lesions (33%) [20].

While Strauss's original description of electrocoagulation for rectal tumors was for palliation of bowel function, rectal discharge, and prevention of impending obstruction, later reports support its use [16]. In a series of 8 patients, transanal coagulation was performed for localized tumors less than 3 cm in diameter and less than 7 cm from the anal verge. Median operative time was 22 minutes (16–35 min), hospital stay for most patients was 24–72 hours (36 median), and 50% required a second procedure (1 required 3 procedures). After a 9-month to 4-year followup, 2 died of distant disease and 6 remained free of disease [21]. Madden and Kandalaft separately reported patients with medical contraindications to APR undergoing palliative coagulation. 5-year disease-free survival was 35% [18]. Salvati and Rubin had a total of 19 patients undergoing palliative coagulation where mean average survival was 6.8 months and all but 3 avoided colostomy [17]. Unfortunately, no studies directly compare other palliative treatments to electrocoagulation at this time.

### 2.2. Nd:YAG Laser Photocoagulation

While different lasers are available for endoscopic use, the neodymium yttrium argon garnet (Nd:YAG) laser has been the most described for cancerous and precancerous lesions of the rectum. The Nd:YAG laser has a wavelength of 1024 nm and lower power settings result in coagulative necrosis of tissue with subsequent tissue sloughing, whereas higher power settings cause immediate vaporization of neoplastic tissue. Both types of tissue injury are useful in patients with rectal cancer who may have bleeding or obstruction, explaining why the most commonly reported use is for palliation [22]. It should be used after careful patient selection for optimal success. This includes noncircumferential and nonrecurrent tumors, lower T stage, and length of the neoplastic stricture <4 cm. Obstructing tumors typically required a greater number of treatments while bleeding and tenesmus require less [23–25].

#### 2.2.1. Polyps

For colorectal villous adenomas, YAG laser use was described in a series of 85 patients after diathermic snare resection. Patients were included who refused surgery or had contraindications or hazards to surgical resection. Sixty-seven (79%) were treated successfully where patients were treated every 15 days until tumor destruction was complete. Tumor destruction was complete in 77% of patients with lesions of ≥4 cm diameter and in 93% of patients with smaller lesions with axial extension. There were no major complications [26]. Another study showed colorectal adenomas successfully eradicated with YAG laser therapy in 84% of cases with a morbidity and mortality of 5% and 0%, respectively [27].

#### 2.2.2. Malignancy

Benefits of Nd:YAG laser on quality of life were shown for patients suffering from obstruction, diarrhea, rectal bleeding, mucus discharge, or pain secondary to tumor bulk, with 88% success rates [23–25, 27–30]. In a retrospective analysis of 3,505 treatments performed on 1,015 patients, Nd:YAG laser was used for recanalization of inoperable lower gastrointestinal tumors. Subjective improvement in symptoms was achieved in 97%, and 3% and 0.5% overall morbidity and mortality rates were, respectively, achieved [27]. In a series from Singapore of 27 patients, palliation of bleeding (*n* = 17) or obstructing (*n* = 10) was achieved in all patients, with an average of 2 sessions required to control bleeding and only 1 to alleviate obstruction [31]. A larger study of 84 patients had a 92% successful rate for hemostasis, 83% restored luminal patency, and 95% success rate for both [30].

When used for palliation, major complications reported include perforation (7%), stenosis requiring colostomy (4%), and delayed posttreatment bleeding (2%); minor complications were transient stenosis (25%), laser-induced bleeding (13%), and pain and warmth (24%) [32]. Another series by Van Cutsem et al. showed complications of perforation (2.3%), rectovaginal fistula (3.5%), rectocutaneous fistula (1%), transient high grade fever with negative blood culture or sepsis (4.5%), and laser induced hemorrhage (4.5%) [25]. Abscess is also possible (1.7%) [24]. Unfortunately, long term symptom relief is difficult likely due to extraluminal tumor growth, complications of laser therapy, and the poor general condition of the elderly patients. In surviving patients, 82% at 1 month, 51% at 6 months, 41% at 12 months, 25% at 18 months, and 46% at 24 months remained asymptomatic [25].

### 2.3. Argon Plasma Coagulation (APC)

APC is commonly used over laser therapy because of its lower cost, portability, and ease of use. It was first used in open surgical procedures but with the development of flexible tip probes it can now be used endoscopically. APC utilizes the ionization of argon gas by electrocautery to fulgurate without direct tissue contact. Its depth of penetration is less and therefore is preferred over laser treatments for colonic lesions or rectal lesions above the peritoneal reflection. Its use has been proven successful in benign, premalignant, and malignant conditions, especially when compared to laser.

#### 2.3.1. Polyps

APC has successfully been utilized for the nonsurgical management of colorectal polyps and adenomas [33–35]. In a case-control study, Zlatanic et al. reviewed 77 patients with large sessile polyps (>2 cm) who underwent piecemeal polypectomy with and without APC [34]. Thirty patients received APC therapy (40 W, 0.8 L/min flow rate) to the residual adenomatous tissue. Six-month recurrence rate was similar to those undergoing “complete” excision of polyp tissue (46% versus 50%) with higher complications (bleeding and perforation) in the complete excision group [34]. This data prompted a randomized study for large (>1.5 cm) sessile polyps utilizing APC after incomplete piecemeal or snare resection, also showing a decreased adenomatous recurrence (10% APC versus 63% no APC, *P* = 0.02) [35]. In a separate series of 29 patients where 58 colonic polyps were removed with diathermic snare followed by APC, 17 hyperplastic, 26 tubular, 8 tubulovillous, 4 villous adenomas, and 3 inflammatory pseudopolyps were treated. Effective destruction of remnant polyp tissue was obtained in 56 (96.4%) polyps in 27 (93.1%) patients. Characteristics of APC power output as well as size, distal location, and villous texture of the polyp all influenced success [36].

#### 2.3.2. Malignancy

For treatment of colorectal malignancies, mainly for palliative intent, APC has also been successful on both short term and long term. Canard et al. treated 21 patients, 6 during radiotherapy prior to surgery, while the remaining 15 were for palliation. Of those treated for palliation, 5 had stenosis, 8 bleeding, and 2 bleeding and stenosis. All but 2 patients were treated successfully and no other treatments were required [37, 38].

### 2.4. Photodynamic Therapy (PDT)

Photodynamic therapy uses a systemically delivered hematoporphyrin drug that is activated by laser light directed at tumor tissue. Drug activation by a specific wavelength of light produces singlet oxygen species that either destroy blood vessels resulting in tumor necrosis and/or directly cause apoptosis. Because tumors often have increased vascularity and permeability, there can be relatively more drug concentrated within tumors, leading to destruction of more neoplastic tissue than normal tissue. Cutaneous sensitivity has been reported; therefore avoiding the sunlight is required for a few days after treatment. Because depth of treatment is directly related to depth of light penetration, most studies using photodynamic therapy for rectal cancer focused on the use of this modality as an adjunct to endoscopic resection or for small rectal cancers in nonoperative candidates, with a depth up to 15 mm reported [39, 40]. Reports of its use for colorectal pathology are scarce and mainly for nonoperable patients for palliation.

#### 2.4.1. Polyps

PDT to treat adenomatous polyps was described after other modalities, such as Nd:YAG laser, were used. Eight patients with 9 colorectal villous adenomas (all but 1 had prior Nd:YAG laser treatment), with polyps measuring 1–5 cm long, were treated with PDT using either a hematoporphyrin derivative or Photofrin as photosensitizer. Multiple (4–16) applications of interstitial photoirradiation with red light (630 nm, 100 mW × 500 s per application) were used resulting in eradication of 7 adenomas (follow-up 9–56 months, median = 12) on follow-up endoscopy and biopsy. There were no local complications but skin sensitivity to light was seen in one patient. Substantial necrosis was produced in the other two adenomas, but they were not completely destroyed, probably due to inadequate light penetration [41].

#### 2.4.2. Malignancy

In a series of 16 nonoperable patients with colorectal adenocarcinoma, Patrice et al. used PDT for palliative intent for pain, bleeding, and diarrhea [42]. Adverse reactions included pain, skin photosensitization, perforation, and stenosis. At a mean follow-up period of 13.4 months, 50% were successfully treated, 31% partially improved, and 19% had no response. Mean recurrence-free period was 17.4 months and 19 months for the five surviving patients [42]. A phase I/II pilot study of 6 patients with massive advanced rectal cancers showed 5 had clinical and radiographic responses to PDT and 1 suffered sunburn after discharge [43]. Palliation for both bleeding and constipation has also been shown, with improvement of symptoms in 7 of 10 patients [40].

### 2.5. Radiofrequency Ablation (RFA)

Radiofrequency ablation (RFA) utilizes an alternating current causing frictional heating of tissues, resulting in thermal injury and coagulative necrosis. While RFA is used commonly for premalignant diseases of the upper gastrointestinal tract, use for rectal diseases is limited and mainly palliative for pain. Other common uses are treatment of remaining disease after surgical resection with tumor fixation to the pelvic side wall or sacroiliac joint where patients are otherwise deemed unresectable [44, 45].

#### 2.5.1. Malignancy

Percutaneous RFA utilized for palliation for recurrent rectal tumors has been described with mixed results [46–49]. In a review of 10 patients with 14 lesions, Ohhigashi and Watanabe used RFA for solitary recurrent tumors in 4 patients while all other tumors were treated with palliative intent secondary to distant metastases [48]. Complications of RFA treatment included abscess (2 patients), neuralgia (2 patients), and bleeding (1 patient). Of the patients treated curatively, 1 recurred (followup 6–36 months) and no patient was palliated successfully. Poor results were thought to be secondary to tumor sizes >4 cm [48]. More successful results for pain using CT guidance of RFA for locally recurrent tumors, located either centrally or peripherally in the pelvis, have been seen. Complete tumor necrosis rates of 78% were achieved and if tumor response was partial second ablations achieved a 66% success rate of pain palliation (no complete necrosis was achieved) [47]. In a series of 8 patients, 6 with rectal adenocarcinoma and 2 with sarcoma, 75% had minor pain, 50% sciatic nerve irritation, 25% ureteric obstruction requiring stenting, and 13% colovesical fistula requiring ileal urinary diversion, but all patients were pain-free at 2 months [49].

### 2.6. Endoluminal Radiotherapy (Endoluminal Contact Radiotherapy)

Chaoul, in Germany in 1939, was the first to develop a contact X-ray tube to treat uterine cervix carcinoma [50]. It was Papillon who popularized contact X-ray therapy for the conservative treatment of rectal cancer in Lyon between 1950 and 1990 [51]. Using a specialized 29 mm proctoscope that allows an X-ray tube to be passed through it for direct contact with the tumor, radiation can be given. Radiation is typically given in fractions of 2000 to 4000 cGy to a 3 cm diameter area (two overlapping fields can be treated) every 1 to 4 weeks for a total dose of up to 15,000 cGy. The advantage of contact radiotherapy to external beam radiation is that a high dose of radiation can be delivered directly to the tumor, minimizing the radiation exposure to healthy tissue [15]. Because radiation is typically used for malignancies, there has been no described role for treatments of polyps. Its use was described for both curative and palliative purposes. Most common side effects include diarrhea (54%), bleeding (37%), pain/irritation/discomfort (26%), and ulceration (23%) within 90 days after treatment and rectal bleeding (51%), diarrhea (40%), and ulceration (12%) after 90 days [52].

#### 2.6.1. Malignancy

Endoluminal radiotherapy is typically used for early stage tumors, T1-2 and N0 tumors, but persistent disease and high local recurrence rates creating a 27% failure rate make its use compared to surgical resection debatable [53]. Tumor characteristics for successful endoluminal radiotherapy include well to moderate differentiation without evidence of extension beyond the rectal wall, no more than 10 cm above the dentate line so that the treatment proctoscope can reach the cancer, and no larger than 3–5 cm because the diameter of the treatment proctoscope is 3 cm (3–5-cm tumors may be encompassed by two overlapping fields) [52]. Using these criteria, Lavertu et al. showed a 76% actuarial local disease control at 5 and 10 years [52]. Ulcerated tumors had a higher local recurrence rate and reduced time to recurrence compared to sessile or polypoid tumors [54]. Using transrectal ultrasonography, the local control rate with endoluminal radiation alone was 100% for uT1 lesions, 85% (90% with no evidence of disease after salvage) for freely mobile uT2 lesions, and 56% (67% with no evidence of disease after salvage) for uT3 lesions and uT2 lesions that were not freely mobile with better results found if the tumor was a uT1 or debulked to only scar [55]. In a series of patients treated from 20 to 155 Gy for both curative and/or palliative intents, better results were achieved with a 5-year local control rate of 89% and 5-year survival rate of 76% in the curative group. Twenty percent suffered from rectal ulceration [56]. In a larger series of 126 patients treated for cure, 71% were free of disease after one treatment and 11% after a repeat treatment. Time to recurrence was 16.1 (range, 1–56) months and 29% of patients recurred and 27% were distant [57].

### 2.7. Other Technologies

Cryoablation is a process that uses extreme cold to destroy or damage tissue. Irreversible electroporation (IRE) uses electroporation to induce permeabilization of cell membranes, increasing the threshold where permeabilization becomes irreversible. While leaving the extracellular matrix intact, it can cause cell death. Benefits of IRE include it is nonthermal, so it is not sensitive to cooling effects of vascular structures, and it is able to preserve important structures such as blood vessels. While other technologies such as cryoablation and irreversible electroporation have been used in the treatment of malignancy, including colorectal metastases to the liver, their use has not been well described up to this date for the treatment of either rectal polyps or cancer. Experience at our institution for IRE continues to be experimental where it is mainly used for the treatment of areas of recurrent/unresectable tumor and for margin attenuation.

## 3. Conclusion

Local ablative therapies for colorectal polyps and malignancies can be used successfully for palliation, but treatment for cure should be used only in carefully selected patients unable to tolerate standard care therapies. Whether they directly compare to local excision or more radical resection has not been proven. Treatments tend to be most successful for polyps and small, superficial, and noncircumferential primary tumors. Of course when local treatment failures occur, surgical resection remains the standard of care with radiation and/or chemotherapy as indicated. For advanced cancers or lesions in the inoperable patient, the benefit of local ablative therapies combined with systemic and radiation therapy for palliation has also been supported.
